# Early childbearing experiences of young university student‐parents in South Africa: A qualitative study

**DOI:** 10.1002/puh2.51

**Published:** 2023-01-12

**Authors:** Seluleko Eric Ngcobo, Kemist Shumba

**Affiliations:** ^1^ Department of Social Sciences, Faculty of Humanities and Social Sciences Oxford Brookes University Oxford UK; ^2^ Discipline of Health Promotion School of Applied Human Sciences University of KwaZulu‐Natal Durban South Africa

**Keywords:** contraceptives, early childbearing, sexual and reproductive health, South Africa, young parents

## Abstract

**Background:**

Early childbearing is a major concern especially in developing countries. This study sought to understand student‐parents’ perspectives of early childbearing, describe their individual experiences and identify the types of support required for such young parents.

**Methods:**

This was an exploratory qualitative study with semi‐structured in‐depth interviews among 20 purposively selected participants. All the participants were young parent‐students at the University of KwaZulu‐Natal in Durban, South Africa. The interviews were conducted in 2018.

**Results:**

The young parent‐students identified a lack of adequate information and knowledge about safe sex, contraceptive methods and prevention of pregnancies that led to early childbearing. The common misinformation that prevented the adoption of contraception methods were societal beliefs among men promoting early childbearing, coupled with the negative attitudes of healthcare workers in providing adequate information and reproductive health services. Both male and female parents suffered life‐changing consequences after early childbearing leading to financial burdens to support their new‐born and the interruption of their education. Government financial schemes and bursaries helped these young parents to financially support their new‐born, whereas family support in terms of taking care of the new‐born allowed them to continue with education. In addition, most participants identified having a child as a motivation for pursuing higher education. There is a need for Life Orientation programmes to be tailor‐made to provide adequate knowledge and sensitise the youth on appropriate life choices and access to contraceptives.

**Conclusion:**

Early childbearing resulted from a lack of adequate knowledge on safe sex and contraceptive methods, poor access to contraceptives, the negative attitudes of healthcare workers in proving adequate sexual and reproductive health services to the youth. Schemes to financially support the young parents and support from family members were key to helping the young parents to continue with university education.

## INTRODUCTION

The United Nations estimates that 15% of young girls become pregnant before the age of 18 years, with a higher burden in low‐income regions [1]. In southern Africa, South Africa experiences disproportionately high levels of early childbearing, which remains a major social concern [4, 5]. Approximately 28% of young women have their first child before the age of 20 years. Most pregnancies are unintended and unplanned, occurring outside of marriage [2]. Despite the evidence that early childbearing among young people is declining in South Africa, the figures remain unacceptably high [3]. A study analysing longitudinal public sector data in South Africa (2017–2021) on teenage pregnancies and birth, early childbirths among girls as young as 10–14 years increased by 49%, whereas pregnancies to girls between the ages of 15 and 19 increased by 18% [5, 6].

Several studies have identified various drivers of early childbearing in South Africa. In a study among school youth, poverty was identified as both a cause and an outcome of early childbearing [4, 6, 7]. Low socioeconomic status, early sexual debut [8, 9], lack of access to, and use of contraception and pregnancy termination, poor knowledge of contraceptives [6, 7], coerced sexual intercourse and age–disparate relationships characterised by power differentials that make women powerless in decision‐making were identified as factors leading to early childbearing. In addition, restricted access to healthcare centres due to health service providers’ negative attitudes towards teenagers seeking sexual health products and services exacerbates early childbearing [8, 10–12].

The United Nations Economic Commission has stated that the high levels of teenage pregnancies remain a major social and economic problem in undeveloped countries [5]. With early childbearing, the household dependency ratio grows, as both the parent and the child are dependent on other family members [2]. This in turn increases the country's dependency ratio, increasing the country's expenditure on social support at the expense of investment in health and education spending among other priorities [6].

Young parents experience the consequences of early childbearing, such as stress, reproductive health issues and dropping out of school. These issues combined undermine the first, third and fourth global strategic sustainable development goals (SDGs), which aim to promote poverty alleviation, increase access to and use of healthcare facilities and facilitate access to quality education [7]. To address this issue, the government of South Africa has introduced several initiatives such as access to free contraceptives to children 12 years and older, not requiring parental consent for accessing contraceptives and mandatory Life Orientation (LO) lessons on sexual and reproductive health, right to contraception and abortion for students in Grade 7 up to Matric level [8]. However, early childbearing remains a common problem. This study was conducted among student‐parents undergoing university‐level education to explore the factors leading to early childbearing, problems around early childbearing and the support required to address those problems.

## RESEARCH METHODS

### Study design and population

An exploratory study using a qualitative approach with semi‐structured interviews was adopted to elucidate the views of 20 student‐parents on their experiences of early childbearing and from 3 key informants on their knowledge and understanding of early childbearing. Participants were registered full‐time undergraduate students at the Howard College campus, University of KwaZulu‐Natal (UKZN). This study was conducted among Black African students in 2018. The UKZN is a conventional higher education institution in South Africa. It comprises five campuses in two cities, Durban, the economic hub of the province of KwaZulu‐Natal, and Pietermaritzburg, which is the provincial capital city. Most students at the institution are Black Africans, mostly isiZulu speaking, Indians and Whites. The study was conducted at Howard College campus in Durban. The number of participants was guided by data saturation, a process wherein data collection stops when participants start to repeat the same themes and narratives such that no new data emerged [20]. The three‐key informants helped to identify information‐rich participants. Those participants that were identified by the key informants also suggested other participants that met the inclusion criteria through snowball sampling [20].

### Study tool and data collection

Data were collected using an interview guide that was generated based on a thorough literature search which was conducted to establish research gaps. A pilot study was done to improve the interview guide. A sample that had similar characteristics with the target sample was used. Data from the pilot study participants were excluded from the main study. The questions on the interview guide were supplemented with prompts and probes to seek clarification on the various issues that emerged.

The participants were approached face‐to‐face, and all the participants that were invited agreed to participate in the study. No participant withdrew from the study. These semi‐structured in‐depth interviews were conducted with an equal proportion from each sex category. Each interview lasted between 40 and 60 min, allowing for rapport development and openness in the sharing of experiences. The interviews were done in June and July of 2018. The interviews were audio recorded using a digital audio recorder, after obtaining their consent. Subsequently, the transcription was done by the first author after each interview. This helped to identify gaps that were filled in subsequent interviews. Interviews were conducted by the first author. The interviews were conducted at the UKZN premises, and only the researcher and participant were present at any given time. The interviewer has ample experience in the practice of qualitative research. The researcher made efforts to build rapport by regularly communicating with both male and female participants prior to conducting the interviews. We acknowledge that the researcher's gender could have influenced rapport and affected the quality of data, especially that extracted from the opposite sex. This is a possible limitation of the study.

### Data analysis

Data were analysed using thematic analysis [8]. Both authors analysed and manually coded the data. Given that different coders were involved, it was imperative to enhance intercoder reliability. Thus, trustworthiness was enhanced through reproducibility across coders, often called intercoder reliability – where the concern is whether different coders would code the same data the same way [23]. Two independent moderators with qualitative research expertise reviewed the coding process and outcomes. No major diversions were observed. It is important to examine coded data to ascertain that phenomenon hidden or embedded in the data is made explicit [23].

### Ethical considerations

The UKZN's Humanities and Social Sciences Research Ethics Committee approved the study (Protocol number: HSS/0600/018M). All the routine social research protocols were duly observed. These were inclusive of, but not limited to voluntary participation, informed consent, anonymity, confidentiality and the right to withdraw from the study without facing any negative consequences emanating from such a decision [10]. The interviews were conducted in quiet spaces, such as study rooms at the University library, students’ residences and other spaces on campus, often characterised by tranquility. The topic is very private and sensitive; hence, the researcher maintained privacy by using serial numbers.

## RESULTS

We describe the scenario of early childbearing experiences in Durban, South Africa of 20 research participants (Table 1) and from the information gathered from the 3 key informants interviewed for this study.  Results focused on the following six themes: (1) factors leading to early childbearing, (2) misinformation and myths about contraception, (3) unprotected sex signifying trust and commitment in a relationship, (4) early childbearing to prove masculinity, (5) weak sexual and reproductive policy translation and (6) lack of parental support and resilience among young parents.

**TABLE 1:** Characteristics of young parents who had early childbearing experience studying at the Howard College campus, University of KwaZulu‐Natal, South Africa, 2018–2019

Participant no.	Age at interview	Age at childbirth	Year of study	Child's age	Primary caregiver of the child
	**Young mothers**				
1	24 years	19 years	2nd	4 years	Participant's mother
2	21 years	18 years	2nd	2 years	Participant's sister
3	21 years	19 years	1st	2 years	Participant's mother
4	18 years	17 years	1st	1 years	Participant's mother
5	24 years	19 years	2nd	4 years	Participant's mother
6	19 years	19 years	1st	2 weeks	Participant's mother
7	20 years	17 years	1st	3 years	Father's mother
8	18 years	16 years	1st	1 year	Participant's mother
9	20 years	15 years	2nd	4 years	Participant's mother
10	19 years	17 years	2nd	2 years	Father's mother
	**Young fathers**				
11	24 years	20 years	2nd	3 years	Participant's mother
12	23 years	19 years	3rd	5 years	Participant's mother
13	19 years	19 years	2nd	4 months	Child's mother
14	23 years	20 years	2nd	3 years	Mother's mother
15	24 years	19 years	3rd	5 years	Child's mother
16	20 years	19 years	2nd	9 months	Participant's mother
17	22 years	19 years	1st	2 years	Mother's mother
18	24 years	18 years	3rd	6 years	Child's mother
19	23 years	18 years	3rd	5 years	Mother's mother
20	20 years	20 years	3rd	3 months	Participant's grandmother

### Factors leading to early childbearing

The findings showed that unprotected sex involving young people is common in South Africa.

“Children at 12, 13, and 14 years of age start dating and have sex without condoms.” (Participant 12, male, 23 years)

The consequences of early sexual debut include unintended teenage pregnancy and sexually transmitted infections, including HIV/AIDS. None of the participants had either planned or considered the implications of unprotected sex, resulting in pregnancy. This often culminated in both unplanned and unwanted pregnancies.

“When you have sex at an early age, you don't know a lot about safe sex, so you end up impregnating your partner.” (Participant 12, male, 23 years)

### Misinformation and myths about contraception

The causes of early childbearing are relatively similar for both boys and girls. It emerged that despite being aware of basic contraceptive methods, such as condoms, injections and oral pills, young people choose not to use them for various reasons. For example, some shunned condoms because of concerns that they reduce sexual pleasure.

“Sex with a condom is like you're not having sex; you actually feel the plastic. If you go skin‐to‐skin, there are particular feelings that are activated. I say ‘No’ to condoms.” (Participant 7, female, 20 years)

Lack of in‐depth knowledge was cited by most student‐parents as one main reason for the non‐use of contraceptive methods. Despite participants’ reproductive and contraceptive knowledge, they did not have detailed knowledge of the various contraceptive methods. Consequently, participants showed high vulnerability to misinformation and myths about contraception. It emerged that young women often refrain from using contraceptives such as injectables because of such fears as bodily fluid gain, weight gain, loose muscles, looking older than one's age and the risk of sterility.

“Injectable contraceptives allegedly fill your body with water and makes you look older in appearance. Importantly, prolonged use of the injection can result in failure to conceive.” (Participant 3, female, 21 years)

False beliefs concerning sterility prevented the use of contraceptive methods. It was widely believed that the use of contraception before having the first child may lead to infertility. The desire to have children at some later stage culminated in contraception non‐use.

“It is believed that if you start using contraception before you have a child, you will ever be able to bear a child.” (Participant 14, male, 23 years)

Female participants also held similar views.

“There is a risk of not being able to conceive as a result of contraceptive use.” – (Participant 7, female, 20 years)

### Unprotected sex signifying trust and commitment in a relationship

Student‐parents reported that aspiring for long‐term relationships with their partners was a common goal for many. A long‐lasting relationship was considered one characterised by trust, which prompted inconsistent use of condoms. Some even reported never using condoms because they trusted their partners and hoped to take their relationships to another level. Condom use was considered a sign of mistrust and non‐use of barrier contraceptives such as condoms was regarded as an affirmation of trust. Trust was taken as being synonymous with an HIV‐negative status.

“When you have sex for a very long time with one person, you ultimately give‐up on condoms. If your partner loves you, you won't sleep with them with a condom. If you love each other, you should touch skin‐to‐skin.” (Participant 11, male, 24 years)

### Early childbearing to prove masculinity

Young fathers reported concerns that society exerts pressure on young men, which compels them to consider early sexual debut. They feared being labelled as lacking in terms of the requisite skills to establish sexual relationships and being infertile, especially when boys younger than them become fathers. This finding shows that young people are often compelled by external pressure to prove their masculinity by having a child(ren).

“People will not understand it if those younger than you become fathers before you. Without such evidence as a child of your own, the ‘Isishimane’ tag is inevitable, and it's embarrassing” (Participant 12, male, 23 years)

*Isishimane* is a derogatory word that is applied to men that are perceived to have difficulties in approaching women for romantic relationships.

Participants mentioned both feminine and masculine roles as contributing to early childbearing. Varying types of sexual coercion that often result in early childbearing were reported by both young men and women. For example, they reported that men often have unprotected sex to “mark one's territory,” which refers to establishing a strong, lasting relationship with a beautiful woman.

“Sometimes it's just done to make sure that your girlfriend stays with you; you impregnate her, which compels her to stay with you.” (Participant 13, male, 19 years)

Young women shared the same view concerning men that trap women in relationships through impregnating them.

“It's clear their babies’ fathers impregnated them deliberately and trapped them. Most of us were very young. The father of my child took my virginity, so somehow when they do that they feel like ‘I have to have this girl in my life forever’. Hence, they leave a mark.” (Participant 10, female, 19 years)

### Weak sexual and reproductive health policy translation

Home, school and healthcare facilities are three institutions that student‐parents felt should have raised their reproductive and contraceptive awareness. Student‐parents felt these three places failed them and constituted part of the causes of their early childbearing. They reported that their teachers were often old, patronising and obsessed with preaching abstinence. The participants stated that LO, one of the compulsory subjects in high school, largely focused on maintaining physical fitness than delving on sex education.

“Our Life Orientation teacher would avoid topics related to sex and sexuality. We would always be in sports grounds doing exercises. Sex and sexuality were taboo.” (Participant 5, female, 24 years)

Young university student‐parents reported that most nurses would often patronise young people who sought such sexual and reproductive health (SRH) items as contraceptives and SRH information. Thus, healthcare workers were accused of being judgemental and rude as they believed serving young females was tantamount to encouraging young people to indulge in sex.

“You're in a clinic and maybe you're still asking for your way around, asking how to get a card, then you hear ‘You're pregnant’ and everyone hears about you.” (Participant 9, female, 20 years)

Some young mothers commented that they had a bad reaction to contraceptives, and when they reported at the clinic, contraceptive methods were not changed. Participants identified continued bleeding as one of the side effects of injectables that resulted in the non‐use of contraceptives. When asked why injectable contraceptives were less common, a participant said,

“I have experienced complications with my periods. I don't want to use contraception anymore.” (Participant 10, female, 19 years)

### Lack of parental support and resilience among young parents

Parents were accused of failing to initiate conversations on sex‐related topics and only encouraged young people to use contraception upon learning that they were pregnant or had impregnated someone. It emerged that most young parents were warned at home not to engage in sex; they were threatened with being thrown out of the home in the event that they either impregnate someone or fall pregnant.

“My mom would turn and say, ‘Dare fall pregnant while young! You will leave my house if you fall pregnant!’ Mothers would shout and scream, no sitting down and talking.” (Participant 9, female, 20 years)

Young parents reported lack of financial support to be a major problem in supporting their children and themselves. Consequently, most of the young parents succumbed to the need to get a job, which resulted in them not having the time and means to continue with their education. Findings indicated that financial support from different sponsors that paid tuition, residence fees, and meal allowances encouraged young people to continue with their education and also allowed them to support their children.

“I get R 1000 [1 United States dollar = ZAR 18.09] monthly through the National Student Financial Aid Scheme (NSFAS). A half of my income goes to my child because my parents are unemployed, I'm the one employed through the student scheme.” (Participant 8, female, 18 years)

The findings indicated that all young mothers were on National Student Financial Aids Scheme (NSFAS), and only 60% of young fathers were on NSFAS, and other sponsors. Monthly stipends that were received ranged from R 1000 to 1250 for NSFAS students across South Africa, and R 1000 to 3000 for other sponsors. Of those funded university student‐parents, their meal allowance was divided into three parts to support themselves, their child and their families.

“When my allowance comes in, I can't just spend my money knowing that I haven't given my child at least R 500.” (Participant 10, female, 19 years)

Young university student‐parents narrated their stories of how they made themselves comfortable with the little money they received from funders. One of the participants was a young father who had a different experience. The participant had a Department of Social Development scholarship, and he was receiving R 2500 monthly allowance during the time of the interview. He gave an account of how he managed his monthly child maintenance as follows:

“Life is difficult, especially when you have twins. When I buy things, I must buy in pairs. If nappies or dippers for R 300 last for a month for singletons, then I need R 600 a month for my twins. I need about R 1500 for two big powdered milk tins and two small ones a month.” (Participant 13, male, 19 years)

Although the young parents were financially responsible for their children, they did not bear the sole responsibility. Most young parents were staying in university residences, and their families and the families of their partners took care of the child. The support from some family members that were rendering free childcare services allowed young parents to pursue their studies.

“I thank my family for being supportive. While I am here at the university, I don't worry about my child's well‐being because they're supporting me; they take care of my child. If my family did not support, I would not be here at varsity studying.” (Participant 1, female, 24 years)

Financial and social support allowed young student‐parents to persevere in their education. All the participants strongly felt and expressed that their children were the sources of their motivation to pursue studies. Hence, they were not just studying for themselves, but wanted to improve the future of their children. One participant said,

“I have a huge load on my back, but at the same time, my child is a huge motivation for me.” (Participant 12, male, 23 years)

## DISCUSSION

The study describes the perspectives and experiences of young student‐parents in the context of early childbearing. The perspectives of young mothers and fathers are relatively similar with regard to contraceptive non‐use and associated factors. The young parents gave detailed accounts of the factors leading to early childbearing and their experiences of hardships playing their multiple and exclusively different roles of being a child, a parent and a tertiary‐level student [18, 20].

### Outcomes for young parents and their children

Young parents experience varying adverse outcomes caused by early childbearing. The negative outcomes differ for young men and women. Physiological risks, such as obstetric complications, risky preterm birth and low birth weight, are disproportionately high among young mothers [14, 15]. In addition, the risk of the child dying during birth is high [11]. It is identified that emotional and psychological distresses are among the negative outcomes of early childbearing among young fathers, with financial stress being prominent as young fathers struggle to fulfil the role of providing finances for the child [12].

Studies show that young parents are at risk of becoming school dropouts due to early childbearing. First, young mothers are highly likely to drop out or be expelled from school due to pregnancy [13] and are less likely to re‐renter the education system [14]. Second, for young fathers, the juggling of fatherly responsibilities and school was reported to be difficult, with young fathers stating that they felt pressured to find jobs to support their children [15]. In addition, according to the Human Sciences Research Council, young fathers are likely to engage in delinquent behaviours and eventually drop out of school [2]. Thus, the education and future economic prospects of young parents and their children are often compromised, making it difficult for them to escape the poverty trap [13].

### Determinants of early childbearing

It was clear that childbearing was a life‐changing event for all the young parents. Young parents reported a high non‐use of contraception, which led to early childbearing. This trend was evident in previous studies in South Africa, and other less economically developed countries across Africa, Asia and America conducted in 2012 and 2015, respectively [21, 22]. The findings indicated that amongst young people, there was a rampant non‐use of contraception due to various reasons that include but are not limited to the desire to demonstrate one's masculinity, belief that using condoms reduces sexual pleasure, and using the woman's pregnancy to keep her in the relationship. Lack of accurate information on contraceptives plays a key role in early childbearing [15]. Negative beliefs worsened by misinformation about contraception, lack of proper knowledge and unprofessional and unhelpful comments from healthcare workers were reasons given for not using other methods such as injectable contraceptives. False beliefs about different contraceptive methods discouraged young people from using contraceptives. Several studies obtained similar findings that young mothers and young fathers’ beliefs such as contraception making young women fat, producing fluids in their bodies and the fear of infertility, resulting in young women not using injectables [11, 20, 23]. Other reasons were interpersonal, such as being comfortable in a trustworthy relationship, culminating in the decision not to use such contraceptives as condoms [24, 25]. Trusting one's sexual partner and the desire to build a stable relationship were associated with the practice of unsafe sex [16]. Similarly, this study found that most young parents were engaged in unprotected sex and never thought that they would get pregnant. However, in rare occurrences, some young fathers would plan to impregnate their girlfriends to trap them in long‐term relationships and prove their manhood to peers and the wider community.

The study found that even when young parents would like to prevent pregnancies, the attitude of healthcare providers hindered their access to contraceptives [7, 23]. Other reasons for the non‐use of contraceptives were based on personal choices such as dislike for condoms as they were found to make sex less pleasurable [17].

### Impacts of early childbearing

Most young parents had their children while still in high school. Nonetheless, they were able to rejoin the education system and continued beyond secondary school level. Thus, it is clear that being financed to take care of the child can pave the way for young parents’ education. It emerged that all young mothers were funded by NSFAS, and 60% of fathers were funded by NSFAS and other bursary schemes. Young parents reported that the various funding programmes were helpful and significantly contributed to their sustenance and child support. Young parents reported that more than 50% of their meal allowances were directed towards their families and child support. NSFAS provided a place to live, tuition and a monthly allowance (R 1000–1250). Importantly, the monthly allowance was divided between the child and the parent.

In the study, all young parents were not staying with their children. Table 1 shows that most children lived with the mother of the participant, and this was applied to both young mothers and fathers. The Teen Parent Support Initiative report states that young parents supported by parents are likely to continue with their education [19]. The caretaking of the child born to young parents demonstrated the importance of family support in encouraging young people to continue with their education without the need to find a job. Families took care of children irrespective of the child's age. Young student‐parents explained that their children were the primary motivation to pursue further education.

## CONCLUSION

Young mothers and fathers had poor knowledge of safe sex, contraception and sex education leading to early childbearing. Young parents faced the life‐changing consequences of early childbearing, including interruption of education and the attendant financial burden. The availability of financial support through funding schemes and social support from family members provided relief and helped young student‐parents to continue with their tertiary‐level education. There is a need to strengthen programmes aimed at sensitising young people about sexual education and contraception. Researchers need to explore ways and recommend on how to design and implement support to young parents to allow them to pursue their education.

## AUTHOR CONTRIBUTIONS

*Conceptualization; data curation; formal analysis; investigation; methodology; project administration; resources; writing – original draft; writing – review and editing*: Seluleko Eric Ngcobo. *Supervision; writing – review and editing*: Kemist Shumba.

## CONFLICT OF INTEREST

There is no conflict of interest.

## FUNDING INFORMATION

No funding to be reported for this study.

## Data Availability

The data that support the findings of this study are available on request from the corresponding author. The data are not publicly available due to privacy or ethical restrictions.

## References

[1] United Nations Children's Fund . Early Childbearing: Early Childbearing Can Have Severe Consequences for Adolescent Girls. UNCF Data . 2021. Accessed 30 November 2022. Available: https://data.unicef.org/topic/child‐health/adolescent‐health/

[2] HSRC . South African National HIV Prevalence, Incidence, Behaviour and Communication Survey, 2017. HIV/AIDS, STIs and TB . 2018. Accessed 30 November 2022. Available: https://hsrc.ac.za/uploads/pageContent/10779/SABSSM%20V.pdf

[3] StatsSA . Demographic profile of adolescence in South Africa . Statistics South Africa; 2018. Accessed 30 November 2022. Available: http://www.statssa.gov.za/publications/Report%2003‐00‐10/Report%2003‐00‐102016.pdf

[4] United Nations . UNDP Support to the Implementation of the Sustainable Development Goals . UN; 2016. Accessed 30 November 2022. Available: https://www.undp.org/publications/undp‐support‐implementation‐sustainable‐development‐goals

[5] Weeks JR . Population: An Introduction to Concept and Issues. 10th ed. Thomson Wadsworth; 2012.

[6] United Nation Development Programme . Sustainable Development Goals . United Nations; 2015. Accessed 30 November 2022. Available: https://sdgs.un.org/goals

[7] Willan S . A Review of Teenage Pregnancy in South Africa‐ Experience of Schooling and Knowledge and Access to Sexual and Reproductive Health Services . Partners in Sexual Health; 2013. Accessed 30 November 2022. Available: https://www.hst.org.za/publications/NonHST%20Publications/Teenage%20Pregnancy%20in%20South%20Africa%20Final%2010%20May%202013.pdf

[8] Braun V , Clarke V . Using thematic analysis in psychology. Qual Res Psychol. 2006;3(2):77‐101. doi: 10.1191/1478088706qp063oa

[9] Neuman WL . Social Research Methods: Qualitative and Quantitative Approaches. 7th ed. Pearson Education; 2014.

[10] Chen X , Wen SW , Fleming N , Demissie K , Rhoads G , Walker M . Teenage pregnancy and adverse birth outcomes: a large population‐based retrospective cohort study. Int J Epidemiol. 2007;36:68‐375. doi: 10.1093/ije/dyl284 17213208

[11] Van Zyl L , Van der Merwe M , Chigeza S . Adolescent's lived experiences for their pregnancy and parenting in a semi‐rural community in the western cape. Soc Work. 2015;51(2):151‐173. Accessed 30 November 2022. http://www.scielo.org.za/scielo.php?script=sci_arttext&pid=S0037‐80542015000200008

[12] Ramulo M , Pitsoe VJ . Teenage pregnancy in South African schools: challenge, trends and policy issues. Mediterr J Soc Sci. 2013;4(13):755‐760. doi: 10.5901/mjss.2013.v4n13p755

[13] Grant MJ , Hallman KK . Pregnancy‐related school dropout and prior school performance in KwaZulu‐Natal, South Africa. Stud Fam Plann. 2008;39(4):369‐382. doi: 10.1111/j.1728-4465.2008.00181.x 19248721

[14] Chili S , Maharaj P . Becoming a father’: perspectives and experiences of young men in Durban, South Africa. S Afr Rev Sociol. 2015;46(3):28‐44. doi: 10.1080/21528586.2015.1059775

[15] Mturi AJ , Bechuke AL . Challenges of including sex education in the life orientation programme offered by schools: the case of Mahikeng, North West province, South Africa. Afr J Reprod Health. 2019;23(3):134‐148. doi: 10.29063/ajrh2019/v23i3.12 31782638

[16] Mda P , O'Mahony D , Yogeswaran P , Wright G . Knowledge, attitudes and practices about contraception amongst schoolgirls aged 12‐14 years in two schools in King Sabata Dalindyebo Municipality, Eastern cape. Afri JPrm Health Care Fam Med. 2013;5(1):1‐8. doi: 10.4102/phcfm.v5i1.509

[17] Seutlwad L , Peltzer K , Mchunu G , Tutshana B . Contraceptive use and associated factors among South African youth (18‐24 years): a population‐based survey. S Afr J Obstet Gynaecol. 2012;18(2):43‐47. Accessed 30 November 2022. http://hdl.handle.net/20.500.11910/3319

[18] Riordan S . Final Evaluation Report of the Teen Parent Support Initiative. Department of Health and Children, Government of Ireland; 2002. Accessed 30 November 2022. http://hdl.handle.net/10147/42505

[19] Ardington C , Menendez A , Mutevedzi T . Early Childbearing, human capital attainment and mortality risks: evidence from a longitudinal demographic surveillance area in rural‐KwaZulu‐Natal, South Africa. Econ Dev Cult Change. 2015;63(2):281‐317. doi: 10.1086/678983 26028690 PMC4443483

[20] Mjwara N . A Qualitative Study of Early Childbearing: Experiences of Black Women in a South African Township . Unpublished Thesis. University of KwaZulu‐Natal; 2014. Accessed 30 November 2022. https://ukzndspace.ukzn.ac.za/bitstream/handle/10413/13816/Mjwara_Pearl_2014.pdf?sequence=3&isAllowed=y

[21] Mjwara N , Maharaj P . Becoming a mother: perspective and experience of young women in a South African township. Int J Res Interv Care. 2018;20(2):129‐140. doi: 10.1080/13691058.2017.1334963 28628376

[22] Mkhwanazi N . Revisiting the dynamics of early childbearing in South African townships. Int J Res Care. 2014;19(9):1084‐1096. doi: 10.1080/13691058.2014.930512 25005345

[23] Mpatswe W , Maise H , Sebitloane M . Prevalence of repeat pregnancies and associated factors among teenage in KwaZulu‐Natal, South Africa. Int J Gynaecol Obstet. 2016;133(1):152‐155. doi: 10.1016/j.ijgo.2015.09.028 26948340

[24] Panday S , Makiwane M , Ranchod C , Letsoalo T . Teenage Pregnancy in South Africa‐with a Specific Focus on School‐Going Learners . Department of Basic Education; 2009. Accessed 30 November 2022. https://www.gov.za/sites/default/files/gcis_document/201409/dobeteenagepregnancyreport.pdf

[25] Peltzer K , Pengpid S . Contraceptive non‐use and associated factors among university students in 22 countries. Afri Health Sci. 2015;15(4):1056‐1064. doi: 10.4314/ahs.v15i4.2 PMC4765412 26958004

